# Complexity in Mathematical Models of Public Health Policies: A Guide for Consumers of Models

**DOI:** 10.1371/journal.pmed.1001540

**Published:** 2013-10-29

**Authors:** Sanjay Basu, Jason Andrews

**Affiliations:** 1 Prevention Research Center, Stanford University, Stanford, California, United States of America; 2 Centers for Health Policy, Primary Care and Outcomes Research, Stanford University, Stanford, California, United States of America; 3 Center for Poverty and Inequality, Stanford University, Stanford, California, United States of America; 4 Department of Public Health and Policy, London School of Hygiene & Tropical Medicine, London, United Kingdom; 5 Division of Infectious Diseases, Massachusetts General Hospital and Harvard Medical School, Boston, Massachusetts

## Abstract

Sanjay Basu and colleagues explain how models are increasingly used to inform public health policy yet readers may struggle to evaluate the quality of models. All models require simplifying assumptions, and there are tradeoffs between creating models that are more “realistic” versus those that are grounded in more solid data. Indeed, complex models are not necessarily more accurate or reliable simply because they can more easily fit real-world data than simpler models can.

*Please see later in the article for the Editors' Summary*

**Summary Points**

- Mathematical models are increasingly used to inform public health policy, but a major dilemma faced by readers is how to evaluate the quality of models.
- All models require simplifying assumptions, and there are tradeoffs between creating models that are more “realistic” versus those that are grounded in more well-characterized data on the behavior of disease processes.
- Complex models are not necessarily more accurate or reliable simply because they can more easily fit real-world data than simpler models; complex models can suffer parameter estimation problems that can be difficult to detect and often cannot be fixed by “calibrating” models to external data. Conversely, complexity can be important to include when uncertain factors are central to a disease process or research question.
- In many cases, alternative model structures can appear reasonable for the same policy problem. Sensitivity analyses not only around parameter values but also using alternative model structures can help determine which factors are particularly important to disease outcomes of interest. Explicit methods are now available to transparently and objectively compare different model structures.

Mathematical models are now routinely used to inform public health policies. In addition to being useful for theoretical simulations of disease pathogenesis, models can be used to estimate the impact of approaches to control epidemic diseases like pandemic influenza or HIV, as well the health impact and cost-effectiveness of interventions ranging from knee surgeries to new pharmaceuticals [1],[2].

General medical and public health readers face a dilemma when presented with increasingly complex models used for public health policy questions: how do we know whether to trust the results of a model-based analysis, and potentially alter health policies on the basis of those results? Models are valuable for planning interventions that cannot be tested through randomized controlled trials (ethically or practically), simulating the implications of alternative theories about disease pathogenesis or control strategies, and estimating population-wide costs and consequences of public health programs. Since every public health policy decision implicitly involves assumptions, simply avoiding models because they have assumptions is not a logical approach to health policy. For example, even a “simple” policy to vaccinate children against pertussis makes several implicit assumptions: that the vaccine supply will be sufficient to generate herd immunity in the inoculated population, that the human and physical resources needed to administer the vaccine to the needy population are available and affordable, and these resources are distributed in the population in a manner that maximizes benefits while minimizing costs. Modeling forces us to make these assumptions explicit, and to compare how outcomes of interest might change if these assumptions were altered (e.g., How much more might it cost to reach populations that are currently far from health clinics?). Hence, models are highly useful precisely because they make explicit the dilemmas inherent to the public health policy process, helping us to systematically refine our thinking about policies, potentially even before they have been implemented in the real world.

While models are therefore useful for addressing public health policy questions, few consumers of models will be able to comb through all of a model's detailed equations to fully analyze the complex relationships embedded in a given model. Here, we address one specific, common dilemma faced by readers: the question of model choice. How does a modeler choose to represent a disease or public health program in a model, and how do we know whether to trust this representation? As we will illustrate, simply determining whether a model structure appears “realistic” can be misleading. Furthermore, looking at the list of assumptions that went into a given model is also insufficient to answer this model choice question. Counter-intuitively, some models with many simplifying assumptions may actually be more helpful to answer key policy questions than more complex models, as we will illustrate.

## Models Are Becoming More Complex, Presenting New Challenges to Readers

It is rarely the case that one model is obviously “superior” to others for modeling a given policy problem. There are many ways to represent the pathogenesis of a given disease, even one that is well characterized. Alternative models have been constructed to simulate the same policy problem, using the same information; for example, very different models were recently used to simulate the reduction of transmission of HIV due to antiretroviral treatment, as well as the cholera epidemic in Haiti, with differing results [3]–[7]. How can readers compare and contrast the results of these models?

Most readers will recognize that reviewing a model's assumptions is an essential component to answering whether a model might apply to a given scientific question—especially if assumptions strongly contradict available data, or if the assumptions render the model inapplicable to a given policy environment. But a drive to make models more “realistic” has led to increasingly complex models with high levels of detail [8].

This trend toward increasing complexity may allow scientists to address increasingly subtle or complex dimensions of a policy problem, but also poses several potential challenges. First, readers should be aware that increasing the number of variables, or parameters, in a model can produce unintended effects. As shown in Figure 3, the number of factors that are included in a model does not determine how well it will forecast a particular outcome, such as a disease prevalence rate or a cost-effectiveness ratio. Rather, every additional parameter in the model introduces new sources of uncertainty and potential to affect results in non-intuitive ways that may either be useful (the model helps identify a critical issue) or deceptive (the model produces strange behavior that reflects the model structure, not a true aspect of disease pathogenesis).

Figure 2 illustrates the concern graphically, depicting two alternative models of human papillomavirus infection and its progression to cervical cancer. One of the models includes multiple latent states of illness (multiple stages of pre-cancerous lesions), which can progress or regress at rates that are poorly characterized. This more complex model may seem more “realistic,” but the parameters defining the rates of disease progression and regression are so poorly characterized that some choices of the parameter values lead to harmonic oscillations in predicted pre-cancerous disease prevalence that are not true of the disease itself, but simply occur when certain choices of model parameters produce a non-linear interaction that causes strange behavior. This does not mean that all non-linear relationships should be avoided (as most simulation models will involve non-linearity), but rather that complex models must be well-characterized in terms of their behavior before they are used for forecasting or the simulation of disease interventions.

## Dilemmas of Model “Calibration” and “Validation”

While Figure 2 illustrates the irony that adding more variables to a model may actually make a model less “realistic” if its parameters' values or behavior are not well understood, it would seem that ensuring that a model “fits” external data should be a sufficient check on the model's validity. “Calibration” algorithms have been devised to fit large models to data, often allowing modelers to infer the value of parameters that are difficult, if not impossible, to observe in real-world studies [9].

However, there are important limitations to model fitting that readers should be aware of. By varying more parameters to fit data, a more complex model can “overfit” the data—as illustrated in Figure 3; the more complex model in the figure fits the early prevalence data more tightly, but “misses the forest for the trees” by failing to capture just the key aspects of disease pathogenesis that are most relevant to determining the overall prevalence of disease. This occurs because so many parameters can be varied over their range of uncertainty that their inferred or “fitted” values can become overly influenced by noise in the dataset, as illustrated graphically in Figure 3.

Rather than proving that a model is “valid,” fitting a model to data should be thought of as a way to “screen out” a model. That is, if the model can't be fit to data using any reasonable ranges for the parameters, then either the model structure is a poor representation of the actual disease process, or the range of parameter values is far from their real-world values. But fitting is not “proof” that a model is the “correct” one, since there are many models that can reasonably fit the same set of external data [10].

A more difficult problem with fitting models is the issue of “identifiability”: when a large number of model parameters are being fit to a small number of data points, multiple different values can be assigned to each variable. More complex models will almost always fit external data more closely; more variables mean more degrees of freedom—more “wiggle room” among parameter values—to fit external data. Far from improving a model, calibrating too many parameters to too little data can produce several inaccuracies (Box 1).

**Box 1. An Example of the Identifiability Problem**

To illustrate the identifiability problem, consider the following model of HIV. A principal concern is how much transmission occurs during the period of acute HIV infection, before an individual can be detected by standard diagnostics [23],[24]. Suppose we have a model of HIV with just two parameters: the number of infections per month during acute HIV infection and the duration of elevated transmission risk during acute infection. But we only have one data point that tells us that a typical infected person causes six secondary infections during their acute infectious period. The rate of generating infections and the duration of acute disease can be varied over many values to fit this single data point; for instance, the rate of infection might be six per month, and the duration of acute disease 1 month, or 3 and 2, or 1 and 6, respectively, all of which multiply to the same number of secondary infections. This is what is meant by a non-identifiable model: multiple combinations of parameters can generate the same observed data, such that the true values of the parameters cannot be determined by the model and available data.

**Figure 1:**
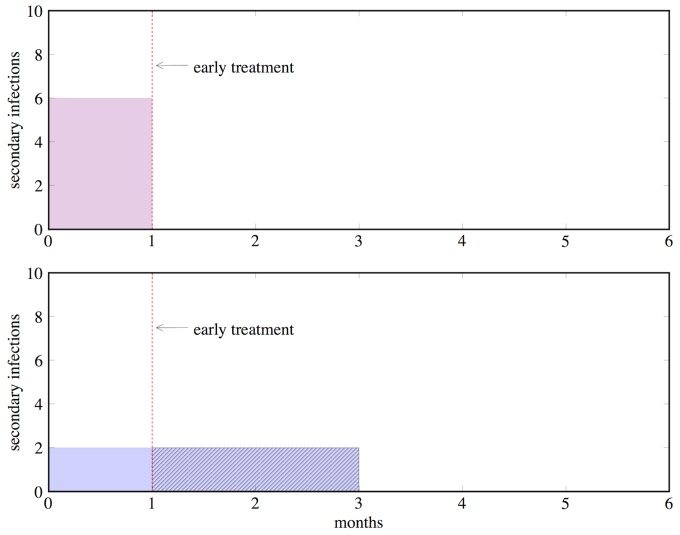
An illustration of the identifiability problem, using an example from HIV policy. Both a 1-month duration of acute infection with six secondary infections per month (top graph) and a 3-month duration of acute infection with two secondary infections per month (bottom graph) produce the same result of six infections per person during the acute infectious period. But the implications of the two different parameter sets are very different, as early treatment (red dashed line) would be effective in preventing secondary infections only in the latter case.

If there were more data to triangulate the parameter values, then we would be able to solve this problem. But since all of these parameters fit equally well to our one data point, the average result from a calibration will simply be the midpoint of the range of parameter values that the modeler specified. For example, if a range of 1 to 5 months were chosen as possible values for the duration of acute infection, the average result would be 3 months at two infections/month. Suppose the true values for the parameters are: duration of 1 month and six infections/month. If our model was simulating an intervention to treat acute HIV (e.g., the “test and treat” strategy of screening for acute infection and giving antiretrovirals at 1 month into the infection [25], or behavioral interventions to reduce sexual risk upon diagnosis [26]), then the true impact on transmission during acute infection would be nil, while the model would project that four infections would be averted (2/month×(3−1) months) as shown in Figure 1. Note that this is a theoretical example, not intended to inform policy on “test and treat” approaches; for further discussion of test and treat details, see [27],[28].

**Figure 2:**
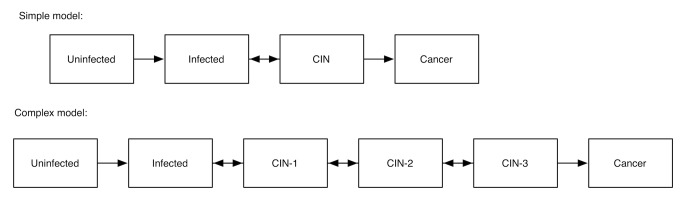
Two alternative models of human papillomavirus and cervical cancer. Pre-cancerous states are designated as cervical intraepithelial neoplasia (CIN) stages 1, 2, and 3.

**Figure 3:**
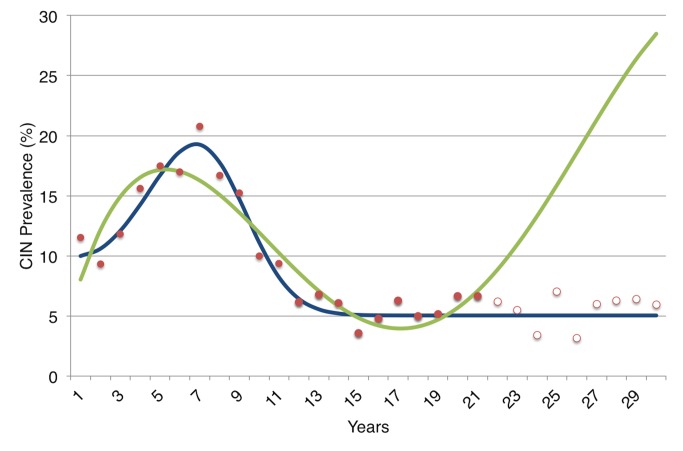
An illustration of the danger of overfitting a model to data in a theoretical demonstration. We first generated data describing the prevalence of all cervical intraepithelial neoplasia (CIN) lesions over a 30-year period among a fictional cohort of young women. To do so, we used the more “realistic” (complex) model in Figure 2 and assigned typical parameter values for the rates of progression and regression between states (a 5% rate of progression to the next state and 50% rate of regression per year to the prior state), then added noise to the data by drawing randomly from a normal distribution with mean equal to average prevalence and standard deviation corresponding to the prevalence rate's standard deviation. We performed a common model “calibration” approach in which both the simple and complex model shown in Figure 2 were fitted to the first 20 years of the data (solid red dots), starting from standard parameter uncertainty ranges for progression and regression of disease [29]. Despite being the “real” model, the more complex model had numerous alternative parameter values fit the data, since there are so many uncertainties about the progression and regression rates that many combinations of parameters were able to produce reasonable fits. As shown, one of these fits (green) produced a pattern that poorly forecast future prevalence (hollow red dots) despite fitting the earlier prevalence data (solid red dots). The more complex model (in green) actually has a better “fit” to the early prevalence data when judged by standard reduced chi-squared criteria than does the simpler model (in blue); but as illustrated here, it has substantially poorer performance in forecasting prevalence in future years. The more complex model did not perform poorly simply by chance; it did so because there was insufficient prior knowledge to inform the parameter values describing the process of progression and regression through pre-cancerous states, hence the model was susceptible to fitting too tightly to the noisy prevalence data (overfitting).

Even computationally intensive “calibration” algorithms that search for millions of possible parameter values to fit a dataset can't overcome the identifiability problem. Because there is not sufficient information to tell which parameter values are more likely to be accurate than others, averaging the results of multiple fits will not work, and sensitivity analyses will be sampling from an infinite range of possibilities (an uninformative result). Many parameters' values can all be fit to data reasonably well, but the mean (or median) of the results will typically be a poor descriptor of the actual parameter space [11]. The recommended approaches to remedy a failure of identifiability are to: (a) return to the field and gather more data to inform the parameter values in the model, (b) use a simpler model that requires fewer parameters if possible, or (c) conduct a theoretical analysis that explores various alternative parameter sets and their potentially different outcomes.

## Sensitivity, Uncertainty, and Model Selection Approaches

When faced with so many uncertainties about the values of parameters and even the structure of models being used to simulate disease, it is common for modeling papers to include sensitivity analyses in which the value of each parameter is varied across its range of possible values. This helps to examine how raising or lowering a parameter's value may raise or lower the value of a model's outcome variable. Similarly, “uncertainty analysis” involves generating error bars around the model's results by sampling from the probability distributions describing the parameter values, examining how variations in the parameter values result in uncertainty around the model's results [12].

However, a common mistake is to assume that sensitivity and uncertainty analyses capture the possible range of results that might occur in the real world. Typical sensitivity and uncertainty analyses involve varying a model's parameter values, not varying the underlying model structure (i.e., the way of representing a disease). Hence, “parameter uncertainty” is captured, but not “structural uncertainty.” Differences in how models are structured can have a greater impact on model projections than differences in parameter values [13]. Variations in the value of a given parameter value could result in a markedly different range of results when that same parameter is input into a different model structure [14].

To address this dilemma, a number of new strategies have been created to perform explicit “model selection”—that is, to generate several alternative model structures and use objective criteria to evaluate which models can best balance complexity and uncertainty (maximizing fit with the fewest parameters, to minimize error). These range from likelihood-based methods that express the probability of the observed data under a particular model, to Bayesian methods that can avoid the complexities of computing a likelihood function for a complex model (such as Markov Chain Monte Carlo methods that select not only parameter values but also “jump” between alternative model structures) [15]–[18]. The strategies all follow one basic principle: that data should inform the level of complexity in a model. If a particular model structure is too simple to address the research question under consideration, then critical variables can be added or alternative model structures chosen so that the disease can be simulated with an appropriately higher degree of complexity. Conversely, if a proposed model is too complex to properly estimate its unknown parameters as relevant to the dataset being used, then the selection method identifies that model as problematic and favors a simpler model. In some instances, a modeler may choose the more complex model because of strong *a priori* beliefs about the necessity of capturing a certain disease or policy process or the finding that a complexity can alter the results in critically informative ways (i.e., the complexity is critical to the question being asked—e.g., in the case of a sexually transmitted disease, the sexual network structure may be critical to ask questions about how heterogeneous contact patterns may influence transmission). In such instances, it should be possible to justify why a more complex model is being utilized. Recent reviews, however, have found that several models can often be employed for the same policy question, using the same data [13],[19]; hence an obviously “optimal” model for a given policy problem may be a rare finding. To date, selection algorithms have not commonly been used in the medical and public health literature [10], and have not been incorporated into guidelines for model reporting [20],[21], even though the approaches have been extensively researched and in some cases automated [10],[16],[22]. While it is much faster to generate one model structure than to undertake the task of comparing alternative models formally, performing explicit model comparisons and selection may be critical to assessing the “robustness” of public health policy modeling results in the future. This would be analogous to the selection of individuals in clinical trials: we require pre-specified, objective criteria for investigators to choose study participants, hence pre-specified, objective criteria can similarly be applied to policy model selection.

## Conclusions

Modelers are usually asked by reviewers and readers to defend simplifying assumptions in models; it would also be reasonable, given the issues discussed here, for reviewers and readers to ask modelers to justify “nonessential complexity” with equal vigor. Models can be treated like computational versions of laboratory experiments—they are meant to explicitly highlight the assumptions that are implicit in health policy proposals, setting up a “clean” analysis to characterize and understand the relationships between key factors affecting health outcomes. Models should, as with laboratory experiments, be sufficiently transparent that their results can be replicated. Models serve as useful tools even when they are simple representations of the real world; new techniques can help us find the right balance between parsimony and realism in an objective manner, using data to build the model from the best available information for any given policy question. As Albert Einstein stated: “Everything should be made as simple as possible, but not simpler.”
